# Integrated On-Chip Transformers: Recent Progress in the Design, Layout, Modeling and Fabrication

**DOI:** 10.3390/s19163535

**Published:** 2019-08-13

**Authors:** Rayan Bajwa, Murat Kaya Yapici

**Affiliations:** 1 Faculty of Engineering and Natural Sciences, Sabanci University, Istanbul 34956, Turkey; 2 Department of Electrical Engineering, University of Washington, Seattle, WA 98195, USA; 3 Sabanci University SUNUM Nanotechnology Research Center, Istanbul 34956, Turkey

**Keywords:** on-chip transformer, CMOS integration, RF-MEMS transformer, monolithic, balun, coupling coefficient, modeling, power gain, *Q*-factor, self-resonance frequency, interleaved layout, stacked layout, transformer characteristic resistance, RFIC, LNA, 5G

## Abstract

On-chip transformers are considered to be the primary components in many RF wireless applications. This paper provides an in-depth review of on-chip transformers, starting with a presentation on the various equivalent circuit models to represent transformer behavior and characterize their performance. Next, a comparative study on the different design and layout strategies is provided, and the fabrication techniques for on-chip implementation of transformers are discussed. The critical performance parameters to characterize on-chip transformers, such as the *Q*-factor, coupling factor (*k*), resonance frequency (*f*_SR_), and others, are discussed with reference to trade-offs in silicon chip real-estate. The performance parameters and area requirements for different types of on-chip transformers are summarized in tabular form and compared. Several techniques for performance enhancement of on-chip transformers, including the different types of micromachining and integration approaches stemming from MEMS (microelectromechanical systems) technologies are also analyzed. Lastly, the different uses and applications of on-chip transformers are discussed to highlight the evolution of on-chip transformer technology over the recent years and provide directions for future work in this field.

## 1. Introduction

With newly emerging wireless communication demands, such as 5G, the realization of fully integrated systems with on-chip components is critical to reduce production costs and overall device size. Along these lines, the integration of many passive devices like inductors, capacitors and transformers is very important, since they serve as the fundamental building blocks of many RF integrated circuits (RFICs) and their performance largely affects the overall circuit specifications [1,2,3,4,5,6,7,8]. Fortunately, the evolution of microelectronic process technologies has allowed, to a large extent, the fabrication of active and passive components on the same chip.

Among the passive components, on-chip transformers are important elements in RF design and they are used in many circuits, including low noise amplifiers (LNAs) [1,2], voltage-controlled oscillators (VCO) [3], impedance matching circuits [9], DC isolation circuits [10], power transfer circuits [11,12] and in baluns for power conversion between single-ended and differential-ended circuits [13,14]. The late 1980s marked the first studies on on-chip transformers [15,16], and since then several groups have investigated the various aspects concerning the design, fabrication, layout, and modeling of on-chip transformers; as well as, their characterization and performance metrics. On-chip monolithic transformers with different turn ratios were first fabricated by Long in 1997 [4,17]. An overall circuit model and characterization of monolithic transformers, which also discusses the analytical formula for calculating self and mutual inductances of on-chip transformers was presented by Mohan et al. in 1998 [18]. Around the same timeline, J.-J. Zhou et al. used monolithic on-chip transformers for fabricating integrated CMOS (complementary metal-oxide-semiconductor) LNAs [19]. The common goal in these studies have been to address the two major limitations in realizing on-chip transformers; which are: (1) Low efficiency mainly due to lossy substrates, and (2) large area occupancy on a semiconductor chip.

In standard CMOS processes, microelectronic devices are fabricated on a substrate material (e.g., silicon, gallium arsenide); however, in case of on-chip transformers, the existence of this underlying substrate severely degrades the performance because of substrate coupling and flux leakage [20]. Due to the challenges in integrating three-dimensional structures (e.g., solenoid) to standard CMOS process technologies, monolithic transformers are mostly built in planar fashion based on an interleaved layout. On the other hand, the planar geometry prevents full confinement of the magnetic field within the device and causes the field to pass through the substrate (e.g., silicon) with finite resistivity which produces magnetic losses. Additional to the substrate losses, there are ohmic losses, due to the thin metallization and parasitic losses between the conducting coil of the transformer and substrate, and also in between the coils of the transformer. 

Despite their lower efficiency, planar transformers are still widely preferred as they are easy to fabricate owing to their simple geometry and ease-of-compatibility with CMOS foundry processes. To achieve better performance, a lot of work has been performed to fabricate stacked-type structures by using thick vias to connect multiple metal layers on top of each other with air gap separation [21,22,23,24,25], which showed better results than simple planar structures. There have also been attempts to utilize MEMS fabrication techniques to build complex, 3D on-chip transformers; despite the process and/or materials related deviations of these approaches from standard CMOS fabrication flows [26,27,28,29]. 

Noting the importance of on-chip transformers in RF systems and the multifaceted issues in their design, fabrication test cycle; this paper provides a comprehensive review and discusses the various attempts that have been proposed to realize on-chip transformers with a detailed comparison of their performance metrics. Comparative study of the various design topologies adopted by different groups in realizing monolithic transformers is described. Previous works on modeling and characterization of monolithic on-chip transformers are described, which includes the different lumped or distributed models for transformers and different analytical formulas for calculating their inductances and other critical parameters. The paper is organized in seven sections as follows: After the introductory Section 1, figures-of-merit for on-chip transformers are discussed in Section 2; in Section 3, different modeling and characterization techniques of monolithic transformers and their comparison are presented; in Section 4, types of CMOS compatible monolithic transformers (either stacked or interleaved) are described in detail; in Section 5, a performance analysis is provided, and different attempts to improve the performance of monolithic transformers are described; Section 6 discusses the applications of monolithic transformers; and finally, in Section 7, conclusion and directions on future work are presented.

## 2. Figures-of-Merit for On-Chip Transformers

To be able to compare the different transformer designs and evaluate their performance, several metrics have been used. In its simplest form, a transformer can be considered as two inductors coupled together [30,31]; therefore, fundamental performance parameters of transformers are also similar to those of inductors which are primarily, the *Q*-factor (*Q*) and self-resonance frequency (*f*_SR_). Apart from these, there are other parameters which describe the performance of monolithic transformers, including their coupling factor (*k*), mutual inductance (*M*), insertion loss and power gain (*G*), area occupied, and transformer’s characteristic resistance (*TCR*). Each of these factors is inter-related to one another, which creates multiple design trade-offs for monolithic transformers. For example, while keeping the number of turns fixed, designing a transformer with a large footprint (substrate area) would allow a larger width and cross-section for the metal conductors. A larger cross section for the conductors reduces the ohmic losses and improves the *Q*-factor. However, parasitic capacitances also increase, due to the large overlapping area with the substrate, which also causes a decreased bandwidth. Therefore, for achieving high *Q*-factor for planar transformers, one has to sacrifice from the self-resonance frequency and area. Similar trade-off considerations can be elaborated for all other parameters. Therefore, the design of monolithic transformers closely depends on the intended application and performance parameters need to be optimized accordingly. The primary performance indicators or figures-of-merit for on-chip transformers are described in detail as follows.

### 2.1. Q-Factor of Primary and Secondary Coil, Q

Primary and secondary coils of a transformer each have their own *Q*-factors, denoted as *Q*_P_ and *Q*_S_, respectively; and they collectively determine the overall performance of the on-chip transformer. The quality factor of a passive device is of great importance as it indicates all the losses and the efficiency of the device. Therefore, one of the main targets, while designing monolithic transformers is to maximize the *Q*-factor for both of the coils. Generally, the *Q*-factor can be stated as follows [32]:

(1) $$Q\;=\;\frac{{\omega L}_{s}}{R}\;\times \;\mathrm{substrate}\;\mathrm{loss}\;\mathrm{factor}\;\times \;\mathrm{self-resonance}\;\mathrm{factor}$$

Another expression given below frequently appears within the context of on-chip inductors, and it can be adopted to express the quality factor of transformer coils in terms of their Y-parameters [33]:

(2) Q = Im(1Y11)Re(1Y11)=−Im(Y11)Re(Y11).

Essentially, *Q*-factor is the ratio of energy stored in a coil and energy dissipated in its series resistance, and can be improved by decreasing the series resistance. At high frequencies, additional phenomena like the skin effect and proximity effect need to be considered, which set critical limitations on the maximum *Q*-factor of a transformer. On the other hand, substrate loss is likely to be one of the major loss mechanisms in inductive devices, and imposes a fundamental upper boundary on the *Q*-factor. Fabricating the transformer on a higher resistivity substrate, such as glass [34], or by shielding the conductors from the substrate have been shown to increase the *Q*-factor of on-chip transformers [31,35]. 

### 2.2. Coupling Coefficient, k

Coupling coefficient (*k*) is a direct measure of the magnitude of magnetic coupling between the two coils of a transformer and it is an important parameter to evaluate the performance of the transformer. Greater overlap area and tighter packing of the two coils of monolithic transformer results in a larger coupling coefficient. A simple formula for coupling coefficient, *k* is given as follows [25]:

(3) $$k=\frac{M}{\sqrt{L_{P}L_{S}}},$$

where, *M* is the mutual inductance between the two coils, and *L_P_* and *L_S_* are the self-inductances of the coils. The self-inductances depend largely on the length of the coils, so the self-inductance can be considered relatively constant if the length remains fixed [24]. Therefore, the coupling coefficient *k*, is actually the measure of the mutual inductance between primary and secondary coils of a transformer. Stacked transformers exhibit higher coupling coefficients, due to their tight geometry [23,25]; whereas, planar interleaved transformers generally have lower *k* values. There is always a trade-off between the voltage transformation ratio *n* and *k*. For applications requiring large voltage or impedance transformation ratios (n = N_1_/N_2_ = V_1_/V_2_), there should be a large variance in the number of turns of the primary to the secondary coil, which inevitably decreases the overlap area between the coils and increases leakage flux, effectively resulting in lower *k* values. 

### 2.3. Self-Resonance Frequency (f_SR_)

Like inductors, transformers also exhibit resonance at particular frequencies referred to as the self-resonance frequency of the transformer. One of the main design criteria for RF-CMOS monolithic transformers is to make this resonance frequency high to increase the operating bandwidth of the device. Transformers resonate at a particular frequency, due to the presence of parasitic capacitances: (a) Between the substrate and the coil, which is directly proportional to the area of the coil facing the substrate; and (b) in between the two coils of the transformer. Due to these capacitances, transformer resonates at a specific frequency beyond which capacitive effects dominate over the inductive effects. For high-frequency wireless applications, the self-resonance frequency (*f*_SR_) is typically in the order of GHz and can be expressed as:

(4) $$f_{\mathrm{SR}}=\frac{1}{2\pi \sqrt{LC_{P}}},$$

where, *L* is the total inductance of the transformer, including self-inductances and mutual inductances, and *C*_P_ is the total parasitic capacitance existing in the transformer.

### 2.4. Power Gain (G)

In its ideal form, the transformer is a lossless device and delivers exact output power as it receives at its input; however, in practice, there are losses (i.e., insertion losses) which decrease the output power. Hence, power gain (*G*) of a transformer, which is the ratio of output to input power is critical and has been used to characterize the performance of monolithic transformers [24,25,36,37] with the following analytical formula [38]:

(5) $$G=1+2\left(x-\sqrt{x^{2}-x}\right),$$

where *x* refers to:

(6) x=Re(Z11)Re(Z22)−|Re(Z12)|2|Im(Z12)|2+|Re(Z12)|2.

This relation uses the *Z*-parameters of the device to calculate the maximum power gain and is applicable while considering the monolithic transformer as a two-port network. 

### 2.5. Transformer’s Characteristic Resistance (TCR)

Transformer’s characteristic resistance (*TCR*) is another parameter of comparison for on-chip transformers presented by Carrara et al. [39]. It is used to characterize on-chip transformers when they are used as tuned loads. *TCR* incorporates both *Q* and *k* parameters of a transformer to evaluate its behavior, as shown in Equation (7). When used as a tuned load in a circuit, the capacitive reactance of a transformer can balance out the inductive reactance, and the circuit can be loaded purely by the resistive component. This resistive component is also called as the input parallel resistance of a transformer and can be a measure of the *TCR*, which is roughly double the value of the parallel input resistance. *TCR* should be taken into account while designing the geometry and dimensions of a transformer, since maximization of the *TCR* is also helpful to optimize the available output power and gain. The analytical formula for calculating *TCR* is given as follows [39]:

(7) $${TCR\;=\;\omega Q}_{\mathrm{EQ}}L_{\mathrm{EQ}},$$

where, *Q*_EQ_ and *L*_EQ_ are the equivalent quality factor and inductance of a transformer given by:

(8) $$Q_{\mathrm{EQ}}=Q_{1}\frac{k^{2}Q_{1}Q_{2}}{1+k^{2}Q_{1}Q_{2}}{\;,\;L}_{\mathrm{EQ}}=L_{1}\left(1+\frac{1}{Q_{1}^{2}}+k^{2}\frac{Q_{2}}{Q_{1}}\right),$$

and *Q*_1_, *Q*_2_ are the quality factors of the primary and secondary coil. Even though the *TCR* parameter is used by some researchers [25] to characterize the device’s performance, still it is less commonly used amongst all other parameters. 

## 3. Modeling and Characterization of On-Chip Transformers

To achieve the desired transformer performance, proper modeling of on-chip transformers is critical during the design stage, since the modeled parameters allow analytical estimation of performance metrics like the *Q*-factor, mutual inductance, self-resonance frequency and coupling coefficient prior to actual device fabrication. On-chip transformers are usually fabricated on silicon substrates, which initiate a large number of frequency dependent parasitic capacitances and resistances (i.e., at high frequencies due to eddy currents) that are difficult to model [40]. There are multiple factors to consider, and this makes modeling of on-chip transformers a rather challenging task in RF design. 

**Figure 1:**
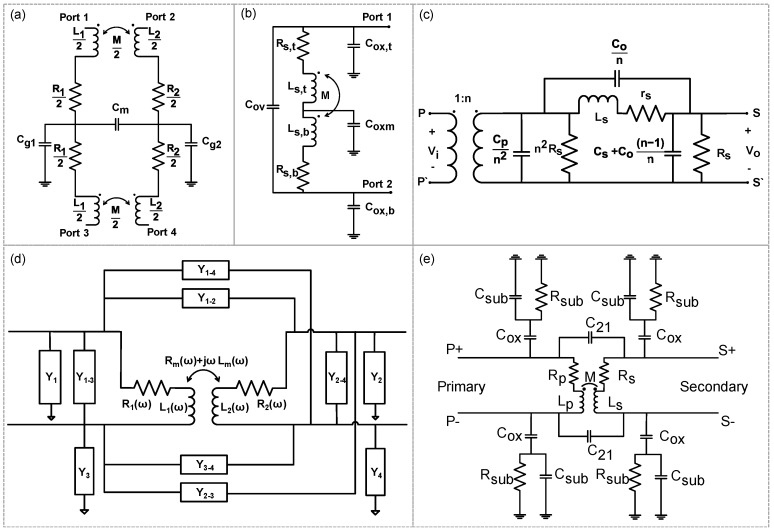
Some examples of earlier circuit models for on-chip transformers, including: (**a**,**b**) Lumped models [15,18] and (**c**) a high frequency model with primary side referred to the secondary [4]. Examples of frequency-dependent on-chip transformer models: (**d**) A compact frequency-dependent model [38]; (**e**) a frequency-dependent model with less error [30].

Several groups have tried to make equivalent circuit models for on-chip transformers. These models for on-chip transformers include lumped designs with discrete RLC elements, as well as the distributed designs with transmission line models. The earliest model for monolithic transformers was provided by Frlan [15] in 1989, which incorporated lumped circuit elements (Figure 1a). The derived analytical expressions proved to be reliable up to 20 GHz when compared with the measured results. 

Another model for on-chip transformers was reported by Mohan [18] (Figure 1b). This is also one of the early models of monolithic transformers developed in 1998, which includes only substrate-to-coil capacitances and coil-to-coil capacitances for a stacked on-chip transformer. In another earlier work, Valkodai and Manku used HSPICE for modeling and simulating thin film inductors and transformers [41]. Essentially, a distributed model was proposed, but still, it was not very efficient for high frequencies above a few GHz. A good high frequency model was provided by Long [17], which took into account the high frequency parasitics (Figure 1c).

At lower frequencies, frequency-dependent mutual resistance and inductance are usually neglected in on-chip transformer models; but, at higher frequencies, they should be taken into account for correct modeling of transformer behavior with less error. In an attempt to establish more accurate circuit models for on-chip transformers, the effects of eddy current losses and skin effect were also taken into account, and a compact model was developed Figure 1d [38]. This model is comprised of frequency-dependent mutual resistance and inductance elements to reflect the frequency-dependent loss mechanisms like the skin effect and proximity effects, such as eddy current losses. To better represent transformer characteristics, an improved model was proposed by Hsu et al. (Figure 1e) [30], which accounts for the effects of substrate coupling and mutual coupling. Using this model, an excellent match was achieved between experimentally measured and calculated parameters.

Along with the compact model, analytical formulas relying on the concept of geometric mean distance (GMD) [42] have also been proposed for fast and accurate calculation of self-inductance values and thereby predict the mutual inductance during the design stage [21,25,43]. An equivalent circuit was drawn, and all the parasitic capacitances and resistive coupling factors were extracted within ~3% accuracy [43]. The analytical formula to determine the mutual inductance of a stacked-type monolithic transformer is given as follows [25,42]:

(9) $$M=\frac{\mu_{0}}{2\pi }l\left\{\ln\left(\frac{1}{d_{m}}+\sqrt{1+\frac{l^{2}}{d_{m}^{2}}}\right)-\sqrt{1+\frac{l^{2}}{d_{m}^{2}}}+\frac{d_{m}}{l}\right\},$$

where, *l* and *d_m_* represent the length of overlap and distance between two coupled coils, respectively.

In another work, a very effective circuit model for planar interleaved on-chip transformers with patterned ground shields (PGS) was proposed. The proposed model was validated for various types of layouts and simple, yet accurate expressions for calculating the self-inductance, mutual inductance, coupling factor, self-resonance frequency, parasitic capacitances and series resistances were described. The accuracy of the model was verified by comparing the calculated and measured results, which only showed a slight difference of ~3.26% [44,45]. Similar methods of modeling and design of on-chip transformers have also been reported [15,46,47,48,49,50,51,52,53,54,55,56].

Progressively, recent advancements in 5G and millimeter wave (mm-wave) networks have also demanded exploitation of on-chip transformers at higher frequencies (i.e., at mm-wave frequency regime) for designing high performance mm-wave integrated circuits. Standard RF design strategies for developing on-chip transformers are not suitable for transformer design in mm-wave domain. For instance, a typical RF design of on-chip transformers utilizes larger metal widths to decrease the series resistance and improve the *Q*-factor of a transformer. Alternatively, smaller metal widths are desirable for transformers at mm-wave frequencies to minimize the substrate capacitive coupling effect, which eventually shifts the self-resonance frequency of a transformer to higher values. Additionally, to improve the *Q*-factor of a transformer at mm-wave frequencies, smaller footprints which possess lower series resistances are usually required [57].

Similarly, appropriate modelling for on-chip transformers at mm-wave frequencies is also crucial for the design of transformer-based mm-wave circuits. However, conventional approaches for modelling on-chip transformers are only validated for frequencies below 30 GHz, and, hence, cannot be applied to mm-wave frequencies (above 30 GHz). Owing to this issue, several attempts have been made in order to model and characterize on-chip transformers at mm-wave frequencies [57,58,59,60,61]. 

**Figure 2:**
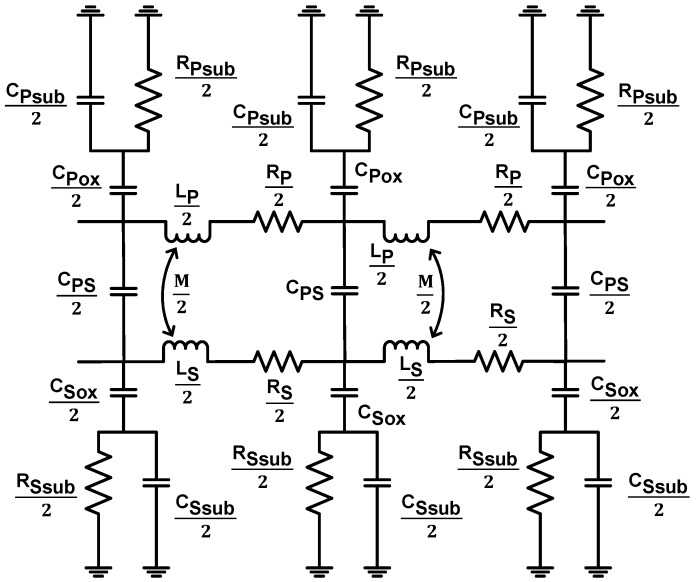
An equivalent model for on-chip transformers based on lumped circuit elements at mm-wave frequency domain [59].

In one study, a lumped circuit model for stacked type on-chip transformers for 60 GHz power amplifier applications was proposed [58]. High frequency parasitic coupling, as well as proximity and skin effects, were carefully modelled using lumped circuit elements, and analytical expressions for model elements were derived and compared with measured results. To achieve high accuracy, the mismatch between the analytical model and measured results was compensated by adding some fitting parameters to the model. This approach provided an accurate model for stacked type on-chip transformers for mm-wave frequencies, and its extension to other transformer topologies could be possible with further modifications. A more generic approach to the model on-chip transformer for frequencies up to 100 GHz in silicon-based CMOS and BiCMOS (bipolar CMOS) technologies using lumped circuit elements were provided by Leite et al. [59] (Figure 2). To ensure its applicability, the model was validated for stacked type transformers with various physical dimensions in two different technologies (65-nm CMOS and 130-nm BiCMOS), and analytical expressions for each element present in the electrical model were derived, which revealed a good overlap between analytically calculated and experimentally measured values.

At higher frequencies (above few GHz), the behavior of a transformer circuit is highly distributive in nature, since the size of the transformer becomes comparable to signal wavelength. Hence, contrary to the lumped circuit element approach, a distributive modelling approach was adopted to model on-chip transformers as coupled transmission lines [60]. Compared to the lumped approach where a large number of lumped circuit elements are required to model high frequency parasitics, distributed modelling provided a simpler equivalent model with fewer parameters [60]. Moreover, modelling of on-chip transformers with center-taps has also been exploited for the design applications of transformer baluns [60,61]. 

## 4. On-Chip Transformers Based on Standard IC Process Flow

One of the main concerns while designing on-chip transformers is to ensure compatibility with standard integrated circuit (IC) process technologies, which is critical to enable monolithic RFIC fabrication. However, IC processes usually adopt in-plane, thin-film metallization steps which force the on-chip transformers to adopt planar topologies as opposed to three-dimensional structures. Such planar topologies create two major drawbacks against the development of on-chip transformers which are: (1) Requirement for large substrate area and increased chip size to accommodate the transformer; and (2) low coupling factors and poor transformer performance. 

To overcome these challenges, different layout configurations have been proposed which can be selected depending on the requirements of the specific application. Two popular configurations used in on-chip transformers are the “interleaved planar layout”, and the “stacked layout” where each topology has its own merits and disadvantages. On-chip transformers based on interleaved planar layouts are simpler to fabricate, but suffer from low coupling factors and occupy larger areas. On the other hand, stacked layouts yield better coupling factor in a smaller area, due to the tight arrangement of transformer coils [62]. But at the same time, they are more complicated to fabricate and cause larger parasitic capacitances because of more overlap among the individual metal layers of the coil and similarly between the metal layers and the substrate. Therefore, the choice of layout directly depends on the application. Details of stacked and interleaved structures for on-chip transformers are provided in the sections below.

### 4.1. Spiral Interleaved Layout

**Figure 3:**
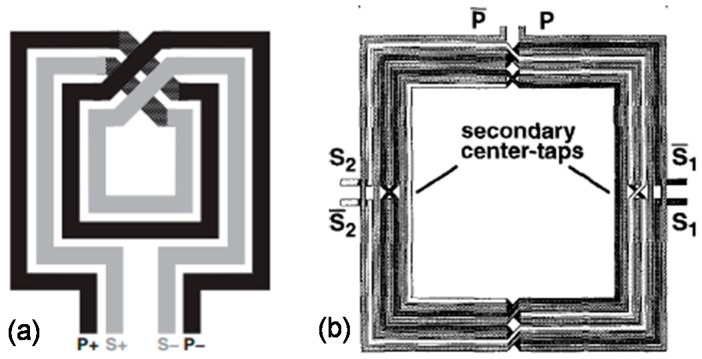
Structure of the interleaved transformer: (**a**) Simple layout of square spiral interleaved two-turn on-chip transformer, reprinted with permission from [63]; (**b**) center-tapped interleaved transformer functioning as a tri-filar balun, reprinted with permission from [4].

Spiral interleaved layout of on-chip transformers suggests that rather than building two planar coils separately on a silicon chip, building them in an interleaved manner with one coil in between the other increases the coupling coefficient of the transformer. A simple layout of square spiral interleaved 2-turn transformer is shown in Figure 3a [63]. Transformer geometries based on the interleaved layout occupy relatively larger areas—and, therefore, they are not efficient in terms of “silicon chip real-estate”. Interleaved transformers are usually designed in 1:1 turn-ratio configuration. In 1:1 turn-ratio configuration, the primary and secondary coils have matching lengths. On the contrary, 1:N or N:1 turn-ratio transformers require coils to have different lengths which cause increased flux leakage, due to poor magnetic coupling between the spiral-based coil geometries laid out in the planar interleaved format. Therefore, transformers with differing turn ratios have smaller coupling coefficients. Despite the lower coupling coefficients, interleaved transformers with different turn ratios have also been used usually in center-tapped configuration, to function as tri-filar baluns which find frequent application in RF blocks like LNAs to convert balanced circuits to unbalanced ones (Figure 3b) [4].

To realize high performance interleaved on-chip transformers, several novel approaches were explored both from the fabrication and the layout aspects. For instance, porous silicon layer (200 µm-thick) was added in between the substrate and the transformer which effectively functioned as a “shield” layer to reduce the parasitic coupling effects between the metal and the substrate [36]. Due to the reduction in parasitics, a huge improvement in self-resonance frequency and gain was observed. In another work, use of the multipath technique with a step-wise increment of the widths of sub-paths (i.e., an inner subpath is narrower than outer one), was employed to decrease the skin and proximity effects [64,65]. Reducing the width of the inner paths helps maintain constant length-to-width ratio, and, hence, constant resistance for each loop, which allows equal distribution of current in all parts of the transformer for better performance. An increase in the coupling factor and gain was observed using this technique for a 3:3 turn-ratio transformer. A similar approach of variable width algorithm was adopted in other studies to determine the optimal metal widths for a planar transformer to achieve minimal resistance [66,67]. As a result, an improvement of ~11.6% in the transformer resistance was observed. Effects of impedance matching on the gain of planar interleaved transformers were also evaluated, and results showed that power gain of on-chip transformers is maximum at specific values of the load impedance [30]. Hence, impedance matching is necessary for applications needing large power gains. Results from various studies are compared and summarized in Table 1.

### 4.2. Stacked Layout

The interleaved structure suffers from low coupling factors and occupies large area. To overcome these problems, the stacked layout technique was introduced for designing on-chip transformers which gives better coupling factors with lower area-budget and at the same time fully-compatible with standard CMOS processes. Indicative of its name, the stacked layout can be realized by placing multiple metal layers on top of each other to make three-dimensional out-of-plane structures which allows better coupling. Tight arrangement of metal coil with each other allows the magnetic flux to be confined within the structure and minimize flux leakage; as a result, better magnetic coupling can be achieved. However, this arrangement also has some drawbacks, which include high parasitics, due to large overlap between metal coils yielding low self-resonance frequency, and relatively difficult fabrication processes compared to planar interleaved structure because of the complex device geometry. 

**Figure 4:**
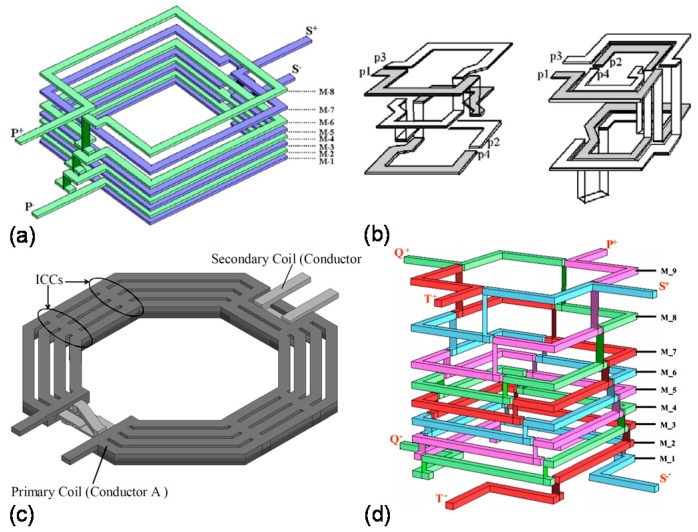
Stacked-type on-chip transformer architectures: (**a**) Three-dimensional view of a stacked layout configuration for on-chip transformers, reprinted with permission from [24]; (**b**) designs involving the “half-coil” concept at each layer with one-turn per layer topology (left) and two-turns per layer topology (right), reprinted with permission from [70]; (**c**) on-chip transformer with high inductance ratio, the inter-coil connections (ICCs) reduce the effective winding inductance and resistance values, reprinted with permission from [71]; (**d**) a stacked layout for on-chip transformers using the “quad-coil” concept, reprinted with permission from [23].

Due to their small area requirement and high coupling factors, stacked on-chip transformers are becoming very popular in RF-IC applications, and a lot of work has been done to maximize their performance. An attempt to make solenoid type vertically-stacked on-chip transformers was presented by Fong et al. [68]. This is one of the very early attempts of making stacked-type on-chip monolithic transformers. Results for this design were impressive showing *k* of more than 0.95, with four times reduction in the area compared to equivalent planar models. Example of a stacked on-chip transformer is shown in Figure 4a [24], where M-1 to M-8 are the metal layers; P and S represent primary and secondary terminals of the device, respectively.

Many models have been proposed to characterize stacked-type transformers [24,25,44], and attempts have been made to achieve high turn-ratio and high coupling factor simultaneously. One example is the use of metal-insulator-metal (MIM) capacitor as the two coils of a transformer to increase its coupling, where *k* of 0.92 with an outer dimension of 140 µm [21] and increased bandwidth with self-resonance frequency reaching 30.8 GHz was achieved. Using negative capacitance (NCAP) to compensate the coupling capacitance between the primary and secondary metal coils has also been shown to improve the high frequency response of on-chip transformer. The results showed the *f*_SR_ value of up to 15.5 GHz, with a return loss of less than −10 dB [69].

Additionally, CMOS compatible stacked on-chip transformers have been developed using the concept of “half-coil” in which, each metal layer assumes two half coil turns corresponding to the primary and secondary sides of the transformer (Figure 4b) [70]. With this approach, coupling coefficients of 0.77 were achieved, and up to 70% reduction in the area was observed compared to on-chip transformer designs in the planar layout. A novel method to achieve high inductance ratio with relatively smaller area for on-chip transformers was presented by Lim et al. [71]. In this method, the primary coil was designed to have a large metal width, and the secondary coil with smaller metal width was routed in between the loops of the primary coil, as shown in Figure 4c. The inductance ratio was observed to be more than 30, and a turn-ratio of 5.8 was obtained using this technique.

There have also been other works which proposed various complex stacked designs to increase the turn-ratio and coupling factor simultaneously [22,24,25]. Most of these designs used the concept of half-turn to build complex stacked structures. Few of these designs were also made with full-coil configuration and stacking each turn of primary and secondary coils over one another. Maximum coupling factor of more than 0.9 and inductance density of 1052 nH/mm^2^ was achieved [24,25].

In some applications like RF wireless transceiver circuits, more than one on-chip transformer may be needed. To address this issue, the concept of “quad-coil” was proposed [23], where each metal layer housed four quarter turns to realize the primary and secondary coils of two transformers (total of four coils) in stacked geometry. Figure 4d shows the quad-coil concept with metal layers M1–M9 and four terminals (P,Q,S,T) to represent each coil. The transformer was implemented by using 90-nm CMOS technology, with an inductance density of 2840 nH/mm^2^ at an area of 0.1 mm^2^. Table 2 above provides a comparative analysis of different stacked-type on-chip transformers based on the various figures-of-merit.

## 5. Design Strategies and MEMS Process Flows to Improve On-Chip Transformer Performance

Due to the inherent trade-off between the competing figures-of-merit, elaborated in Section 2, performance of on-chip transformers should be evaluated by selecting the critical parameters for the target application. One of the major factors which degrades the performance of on-chip transformers is the substrate loss, due to magnetic flux leakage into substrate with finite resistivity, and the capacitive coupling of metallic windings with the substrate. While the parasitic coupling effects are negligible at low frequencies, at higher frequencies they cause severe degradation in device performance. At higher frequencies, the “skin effect” also becomes critical and limits the current to only pass through the outer layer of the conductor with a specific depth (i.e., skin depth), which reduces the effective area of the conductor and results in larger coil resistance. 

All of these losses affect the critical performance indicators, including the *Q*-factor, power gain and insertion loss of the transformer. Therefore, to achieve high performance on-chip transformers several strategies have been explored which primarily focused on minimizing the substrate loss effects by: (a) Including a ground shield in between the device and the substrate, and (b) employing various micromachining techniques inspired from MEMS process flows to fabricate devices which are otherwise not possible with conventional IC process flows. 

Using MEMS fabrication techniques, high quality passive devices can be built, but all other RF circuitry still need to be built using mainstream IC processes. As a result, in most cases, mainstream IC processing is preferable to implement on-chip transformers. Monolithic fabrication of MEMS and CMOS is also one solution for building high-*Q* passive devices, but the additional micromachining processes are likely to increase manufacturing costs, which may go very high, especially in large scale production. There have also been attempts to introduce back-end-of-the-line (BEOL) post-IC processes that allow CMOS compatible integration of high performance MEMS components.

### 5.1. Patterned Ground Shield (PGS)

**Figure 5:**
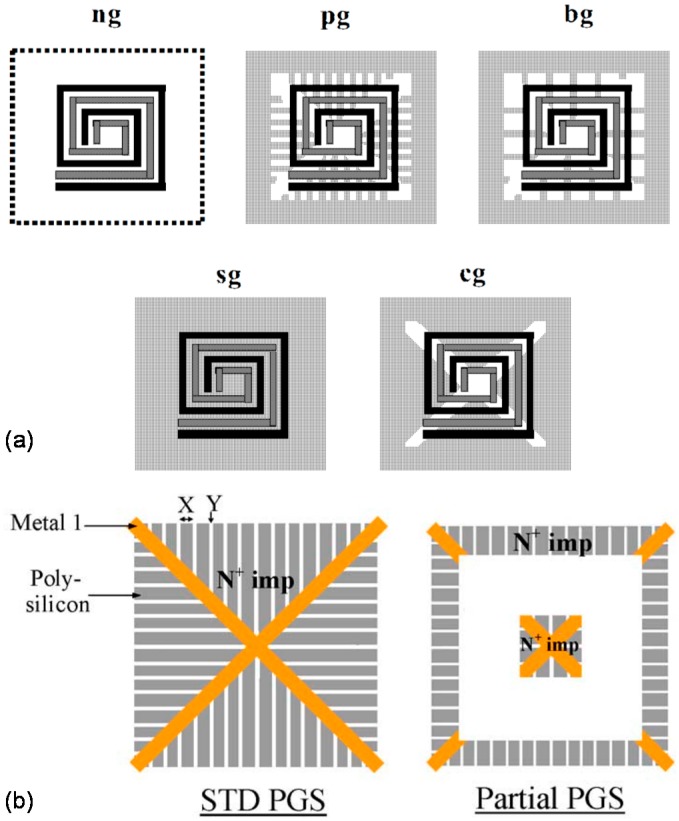
Various types of patterned ground shields: (**a**) Standard, complete patterned ground shield, reprinted with permission from [35]; (**b**) partially patterned ground shields, reprinted with permission from [74].

Ground shielding is one method to improve performance characteristics of on-chip spiral transformers and especially useful when broadband operation is needed. The simple ground shield is essentially a metallic layer which blocks electric field lines from penetrating into silicon substrate and reduces capacitive coupling between the coil and the substrate. As a result of reducing this loss mechanism, which is also referred to as substrate parasitics, the high frequency response of monolithic transformers can be improved. However, due to the electromagnetic field passing through an integral layer of metallic ground, eddy currents are also induced in this layer, which decreases the inductance of the on-chip transformer. Several approaches have suggested patterning of the ground shield (Figure 5a) to reduce eddy currents [20,35], which proved to be effective at high frequencies. The patterned ground shield has also proved to be effective when used in between a primary and secondary metal layer of stacked type on-chip transformer for applications where high isolation is required [72]. Likewise, degradation of transformer performance (e.g., *Q*-factor) with rising temperature can also be compensated to a certain degree with the use of patterned ground shield specifically for interleaved type on-chip transformer designs [73]. Partial patterning of the ground shield was also reported to further increase the *Q*-factor, gain and resonance frequency of the transformer as compared to traditional patterned ground shielding techniques (Figure 5b) [74].

It is critical to note that, although ground shields are helpful at sufficiently high frequencies, to increase the *Q*-factor, gain and sometimes inductance of on-chip transformers; at low frequencies, PGS structures actually lower the *Q*-factor of the transformer. This is due to competing for loss mechanisms in the transformer, in which ohmic losses dominate at low frequencies, and, therefore, placing PGS could further increase losses, due to induced eddy currents in the metallic shield [20].

### 5.2. MEMS Processes and Post-IC Integration Techniques

With the increasing demand on high performance on-chip transformers and evolution of MEMS, building on-chip transformers using MEMS processes is becoming popular, due to the versatility of micromachining techniques and materials in MEMS fabrication. Contrary to standard integrated circuit (IC) fabrication techniques which usually accommodate planar processing of materials and patterning of multiple layers to form stacked structures, MEMS, utilizes additional process capabilities like surface and bulk micromachining, thick photoresist processing, sacrificial layer release techniques and others to achieve complex 3D structures. Since one of the major loss mechanisms for on-chip transformers is concerned with the substrate which has a finite resistivity, by removing the underlying substrate or locating the device far away from the substrate, performance of passive elements can be improved.

**Figure 6:**
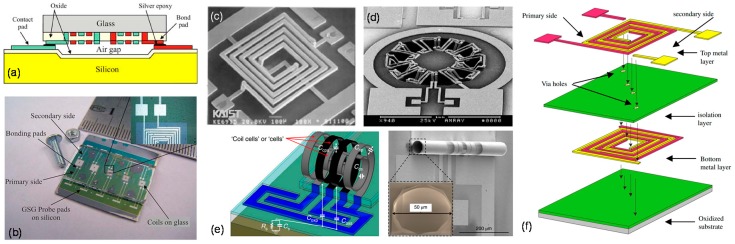
On-chip transformers enabled by MEMS processes and integration techniques: (**a**) Cross-sectional schematic view of a MEMS on-chip transformer using bulk micromachining and wafer bonding and (**b**) image of the fabricated device, reprinted with permission from [34]; (**c**) a surface micromachined MEMS on-chip transformer using thick photoresist process, reprinted with permission from [28]; (**d**) example of a toroidal on-chip transformer realized by bulk micromachining process, reprinted with permission from [32]; (**e**) surface micromachined, roll-up on-chip transformer, reprinted with permission from [76]; (**f**) stacked transformer structure isolated by insulating oxide layer, reprinted with permission from [77].

For instance, bulk micromachining and wafer bonding techniques were used to fabricate on-chip transformers on glass wafers to harness the higher substrate resistivity and reduce eddy currents [34] (Figure 6a). Upon fabrication of the transformers on glass, a trench was formed on a silicon carrier wafer by bulk etching with potassium hydroxide (KOH). Then, the glass substrate was bonded onto silicon using a layer of conductive epoxy, which allowed robust integration of the transformer with the silicon chip (Figure 6b). Similarly, surface micromachining and thick photoresist process were used to fabricate a tall, suspended, spiral monolithic transformer in rectangular geometry [28] (Figure 6c). Toroidal on-chip transformers were also formed using bulk micromachining based on a combination of anisotropic wet etch and isotropic dry etch processes [29,32,75]. Transformers in toroidal geometry displayed higher *Q*-factor of 9.1 and *f*_SR_ of 6.5 GHz, due to reduced magnetic leakage owing to the toroid geometry (Figure 6d).

In another work, the use of front side maskless MEMS process was adopted for improving the performance of monolithic RF-CMOS transformers [27]. By using front side etching as a post-process, substrate underneath was removed, and improvement in *Q*-factor was observed from 0.5 to 6. Due to partial removal of the substrate, parasitics were reduced, *f*_SR_ was increased by 20%, while the gain was increased by 49%. Likewise, on-chip transformer with three-solenoid windings functioning as a balun with improved performance was fabricated based on a CMOS compatible post-process called “concave-suspending micromachining” [14,78]. A more recent effort to realize on-chip transformers with CMOS-compatible processes employed the concept of residual stresses [79] to roll-up planar membranes into a three-dimensional coil geometry [76] (Figure 6e). To improve the performance of the transformer, attempts were also made to incorporate magnetic cores to the on-chip coil [80,81,82], where the magnetic layer was built over the spiral transformer in the form of a bridge structure. The integration of magnetic core was helpful to improve the coupling coefficient of the transformer and to achieve relatively low insertion loss of 1.4 dB [80].

Stacked designs incorporating MEMS processes for the development of on-chip transformers with high coupling coefficients were also reported [77]. Coils were arranged in a stacked order and isolated both from each other and the substrate by insulating layers of silicon oxide (Figure 6f). The two metal layers were connected to each other by via holes patterned in the sandwiched layer of silicon oxide in between. Thick photoresist processing was used to develop thick top metal layer which sufficiently filled the via holes to ensure electrical connectivity between two metal layers. Various types of transformers differing in the manner of overlap between the two metal layers were fabricated and compared, which revealed high coupling coefficient *k* for highly overlapping structures, but lower *Q*-factor due to larger metal-to-metal parasitics. A comparison of the different on-chip transformers enabled by MEMS process flows and/or integration technologies are summarized in Table 3.

### 5.3. On-Chip Transformers with Integrated Magnetic Cores

A common strategy to further improve inductive coupling between transformer windings is by inserting a magnetic core which is straightforward at macro scale. However, at micro scale, due to limitations of standard IC processes to fabricate complex 3-dimensional geometries, realization of monolithic transformers with integrated magnetic cores can be a challenging task. 

**Figure 7:**
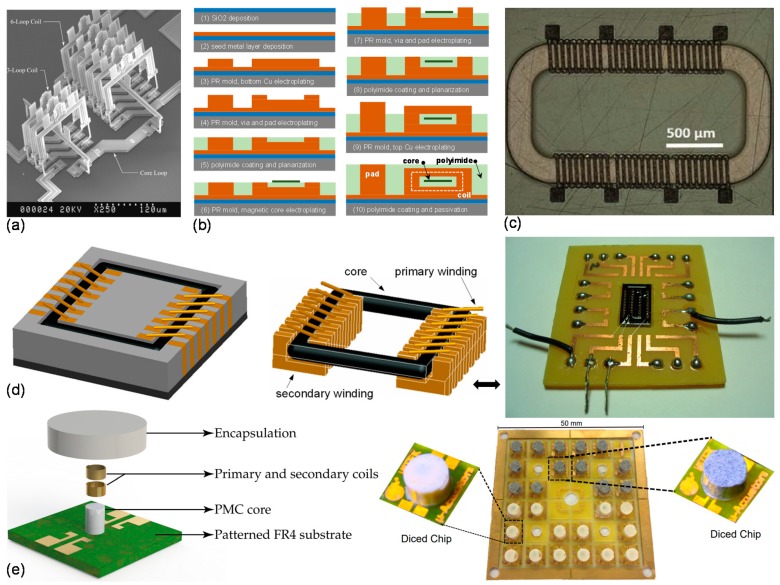
Various approaches to realize microscale transformers with integrated cores: (**a**) Micro-assembly using micro grippers, reprinted with permission from [86]; (**b**) thick resist and electroplating based process flow and (**c**) image of a fabricated transformer with a magnetic core, reprinted with permission from [87]; (**d**) ferrofluid-based core with microchannel [89]; (**e**) polymeric magnetic core using molding, casting and assembly [90].

Considering this issue, there have been attempts to fabricate on-chip transformers with integrated magnetic cores [86,87,88,89,90]. In one study, a micro-assembly system with robotic microgripper was introduced to fabricate out-of-plane 3D microstructures [86]. Initially, the desired microparts were designed and fabricated on a substrate as suspended structures with few weak anchor points referred to as “tethers”. Next, a microgripper was used to hold and free the micro parts from the substrate by breaking from tethers, after which the micro parts are transferred onto another substrate on which assembly is performed to realize the final device structure, including miniature transformers with magnetic cores (Figure 7a) [86]. Although transfer and assembly processes do not offer monolithic fabrication, robotic micromanipulators with precise registration accuracy could be automated to enable efficient assembly and realization of complex 3D structures. 

Another method to insert a magnetic core inside the transformer windings is by using thick photoresist processing and electroplating. In such a fabrication strategy, the transformer windings consist of two layers of conductors between which a magnetic core is sandwiched, and vias are used to connect the top and bottom conductor layers [87] (Figure 7b). With this approach, an on-chip transformer with a magnetic core of 7-µm-thick patterned Permalloy (Ni-Fe) was fabricated, and characterized to exhibit inductance values of 90 µH and 164 µH for primary (10 turns) and secondary windings (18 turns) respectively, for frequencies below 600 kHz [88]. Similar fabrication approach with improved transformer design to realize monolithic transformers with Ni-Fe-W core was also reported where high frequency transformer operation reaching 70 MHz was achieved [87] (Figure 7c). While this fabrication approach offers monolithic integration, the need for the bottom and top conductor layers, magnetic core, and vias necessitate repetition of thick photoresist processing steps, metal seed layer deposition, electroplating, and core-coil insulation which require multiple post-CMOS processing steps and could increase cost and complexity of the overall chip fabrication.

In one study, the idea of using the liquid magnetic core for monolithic transformers was also investigated [89]. The winding design for transformer consisted of two metal layers connected by vias. A sequence of depositing chromium/copper seed layer and electroplating was used to build the bottom metal layers and vias; however, the top metal layer of the conducting coil was fabricated by wire bonding technique [91] (Figure 7d). For the liquid magnetic core, oil-based iron oxide (Fe_3_O_4_) nanofluid was prepared and placed between the metal layers of transformer windings by means of a channel insulated from the conductors with a layer of polyimide. Addition of a magnetic core even in liquid form, clearly revealed improvement in mutual coupling coefficient when compared to equivalent transformers with air core; but, with the expense of higher coil resistance, due to additional magnetization losses of nanofluid core and lower *Q*-factors [89].

For low frequency applications (up to few hundred MHz), use of a polymeric magnetic core (PMC) for microscale transformers fabricated on FR4 substrate was explored [90]. Preparation of PMC involved mixing of powdered magnetic material (NiFeZn) with polymer binding material (e.g., SU8, epoxy or PDMS, etc.). First, an electroplated Au layer was patterned on the substrate to create the pad structures. Next, a Teflon mold was used to build cylindrical polymeric magnetic cores on the substrate by casting technique. Finally, a wire bonding method was used to wind gold coils around cylindrical PMC (Figure 7e). Inductance values of 277 nH and 270 nH were achieved for the primary and secondary windings, respectively, with a coupling factor of 0.96 and footprint area of 0.79 mm^2^ [90]. This approach provided a rapid and low-cost route for realizing microscale transformers. However, the application of the technique specifically on FR4 substrates and its limits on miniaturization, currently prohibit this approach to serve for monolithic fabrication of on-chip transformers on semiconductor substrates.

## 6. Applications of On-Chip Transformers

Transformers have properties similar to that of inductors. As such, they can be used to implement electrical devices like baluns and utilized in various circuits, such as power amplifiers, low noise amplifiers and voltage-controlled oscillators for impedance matching, signal coupling, and phase splitting.

### 6.1. Balun

A major application of on-chip transformers is in the RFIC front-end where they are employed as baluns to achieve coupling between balanced and unbalanced circuits [1,2]. Conventionally, differential amplifiers are commonly used for conversion of single-ended (unbalanced) signals to differential (balanced) ones at RF and microwave frequencies [4]. Alternatively, microwave balun topologies can be used, but occupy a large chip area, especially for designs with target operation frequency below 15 GHz, since their sizes are comparable to the signal wavelength [17]. On the other hand, the size of transformer type baluns are independent of the operating wavelength and can be effectively used for impedance transformation and matching purposes [13]. Transformer baluns can also be used as inter-stage coupling elements in RF mixers to increase their dynamic range [17]. 

**Figure 8:**
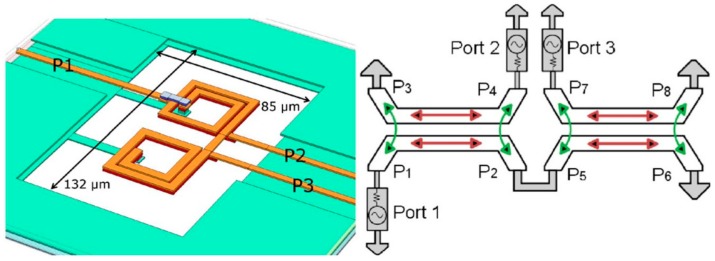
3D layout of on-chip transformer balun with two distinct single-ended secondary windings, reprinted with permission from [13].

A novel approach to develop on-chip transformer baluns in the stacked configuration is based on a design which includes a stack of two consecutive identical spirals connected in series to function as a single-ended coil, and two distinct spirals acting as differential coils correspondingly (Figure 8) [13]. The primary and secondary coils are separated by an insulating layer (600 nm-thick silicon dioxide) where the compact stacked configuration proved to be efficient in terms of area specifications achieving critical feature size of less than 100 µm.

The concept of using on-chip transformer baluns for the conversion of balanced-unbalanced circuits can be extended to mm-wave frequencies as well [61,92]. However, a careful design approach is required, since parasitic capacitive coupling increases at higher frequencies, which causes poor isolation between differential end ports of a transformer balun and limits the mm-wave operation. To address this issue, an isolation circuit can be introduced between the differential ports of the balun to improve the isolation characteristics [92]. 

### 6.2. Low Noise Amplifier (LNA)

**Figure 9:**
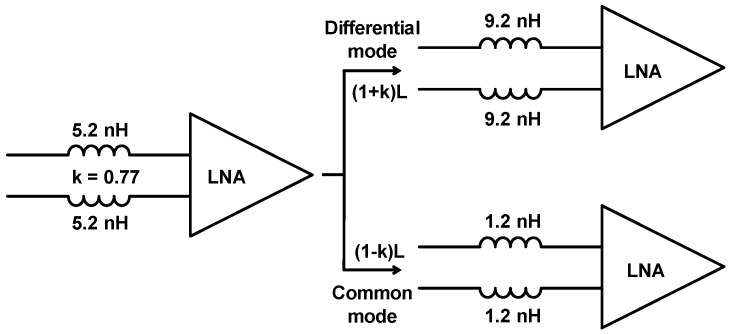
The behavior of a spiral transformer employed in a fully differential low noise amplifier (LNA) circuit will display a (*1* + *k*).*L* factor increase in inductance in differential-mode operation, and (*1* − *k*).*L* factor reduction in inductance in common-mode operation; where *k* stands for the coupling coefficient and *L* is the inductance [2].

On-chip transformers are also adopted in low noise amplifier (LNA) circuits [2,93,94]. An important application is the use of on-chip transformers in differential RF-CMOS low noise amplifier to implement an on-chip tuning network (Figure 9) [2]. Rather than using a pair of inductors, an on-chip transformer was used in this work and results showed improvement of up to 45% in *Q*-factor and 12% in *f*_SR_. The higher performance of a transformer as compared to two separate inductors was attributed to the mutual coupling of transformer windings which increases the effective inductance for the same metal length. Hence, the total length and size requirement on transformer’s metal coils can be reduced (Figure 9). This results in smaller series resistance, shunt capacitance, and die area to be achieved. Along with *Q*-factor improvement, due to reduced series resistance, on-chip transformers can also display advantages in rejection of common-mode signals. For instance, substrate noise coupling, which appears as a common mode signal, due to the symmetric design of the transformer can be mitigated using a lower common-mode gain of the transformer [2]. 

**Figure 10:**
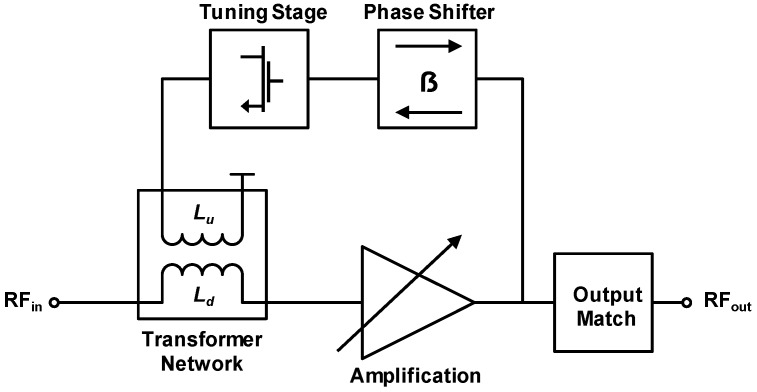
A four-stage LNA design with transformer-based tunable input matching circuit [95].

Recently, there have also been efforts to design transformer-based LNA for software defined radio (SDR) applications [95], where the RF transformer serves as a tunable input matching stage in a four-stage LNA design (Figure 10). Tunability of the transformer matching circuit can be achieved by varying the current in the secondary coil (*L*_d_ in Figure 10) [96], which eventually changes the resonance frequency of the matching circuit. In this way, a tunable LNA with an operating frequency range of 2.2–2.8 GHz was implemented.

### 6.3. Power Amplifier (PA)

Monolithic transformers also assume a key role in the integrated power amplifier (PA) applications, due to their capabilities of DC isolation, network tuning and balanced to unbalanced (balun) signal conversion [97,98,99,100,101,102]. Compared to single-ended power amplifiers, push-pull and balanced power amplifier topologies help mitigate common mode noise, exhibit higher stability, require simpler inter-stage matching circuits and allow cancellation of mismatch reflections from the amplifiers [102]. Specifically, in push-pull architecture impedance doubling can be achieved, which manifest itself as an important benefit in RF PA design [102]. However, the benefits of balanced PA architectures over single-ended topologies bring additional requirements in terms of input and output stage circuitry for balanced to unbalanced signal conversions at the input and output, and this is where transformer baluns come into play [102]. This concept was studied in an early approach which aimed at realizing balanced power amplifier for GHz frequency range based on a two-stage amplifier design coupled with monolithic transformers to serve as input baluns; as well as, input and inter-stage matching circuits [97]. Contrary to traditional PA designs which require additional input DC blocking capacitors and bulky transmission lines for impedance matching, the proposed design proved to be area efficient. 

A similar design approach for multi-stage transformer-coupled power amplifiers (PAs) for mm-wave frequencies was also reported [58,98]. At higher frequencies, substrate losses for transformers increase drastically; therefore, the design of the transformer-coupled RF device can be a challenging task. To address this issue and estimate the high frequency response of the transformer, appropriate mm-wave frequency (60 GHz) models were developed and applied in the design and fabrication of transformer-coupled 60 GHz CMOS power amplifier with a power gain of 5.6 dB and footprint of less than 0.25 mm^2^.

As maximization of the output power is critical for PAs, strategies have been developed to address this need. In one study, a fully integrated 2.4 GHz differential pair class-E power amplifier for Bluetooth applications using on-chip power transformers for signal conversion and load transformation based on standard 0.13 µm CMOS process was demonstrated [5]. Owing to the low voltage specifications of CMOS devices, the overall design was divided into two identical parallel branches of differential amplifiers to decrease the voltage stress on active devices without compromising on the output power. Monolithic transformers were used at the input stage to split and feed the single-ended input signal into two parallel amplifier branches. At the output stage, a similar transformer configuration was used to recombine the amplified signals coming out of the two amplifier branches. This approach provided high output power with power gain of 17.83 dB at 2.4 GHz for 1 V supply [5].

**Figure 11:**
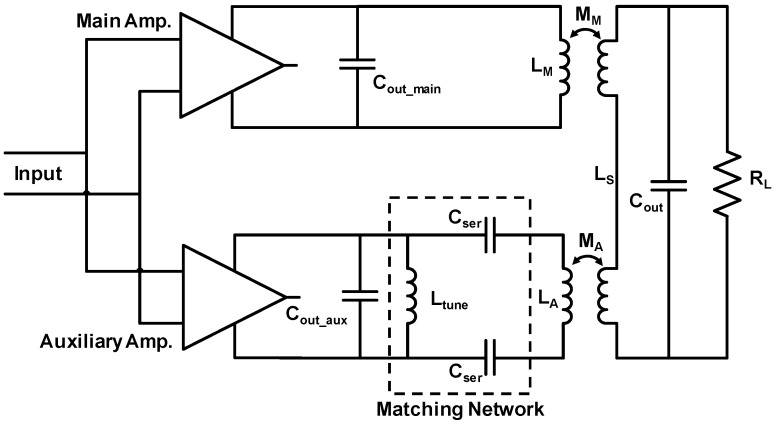
Schematic of an mm-wave Doherty power amplifier with additional asymmetrical transformer matching circuit at the auxiliary side for efficiency enhancement [99].

Another strategy to enhance the output power is based on high frequency (mm-wave) Doherty operation of power amplifiers [99]. Conventional Doherty power amplifier topology contains a parallel combination of two amplifiers (main and auxiliary, where the main amplifier is operational all the time, and auxiliary amplifier turns on only when the output power falls below a certain value) and a transformer-based power combiner at the output. Hence, PAs based on the Doherty topology ensure increased output power; however, the finite output impedance of the auxiliary amplifier (when the auxiliary amplifier is off), due to high frequency parasitics, degrades the overall efficiency of the amplifier system by loading the subsequent power combining transformer at the output. To overcome this challenge, a strategy to include asymmetrical transformer-based matching network at the output of the auxiliary amplifier was suggested (Figure 11) [99]. This additional circuit acts as a matching network to tune out parasitic capacitances when the auxiliary amplifier is off, and, hence, high output impedance is achieved. Alternatively, when the auxiliary transformer is on, the matching circuit acts as a step-down transformer, effectively decreasing the output impedance while increasing the power output [99].

### 6.4. Voltage-Controlled Oscillator (VCO)

Other applications of on-chip transformers include using them in voltage-controlled oscillators (VCOs) [103] because of their higher quality factor as compared to inductors of similar size [3]. In general practice, inductor-based LC tanks are used in VCOs as a resonating circuit. However, replacement of traditional LC tanks with transformer-based resonator circuits coupled with varactors revealed significant improvement in phase noise and sharper impedance response near-resonant frequency as compared to LC tanks [104]. Similarly, different design topologies for low noise VCOs incorporating transformer-based resonators extending up to mm-wave frequencies have been reported [3,103,104,105,106,107].

For instance, the use of on-chip transformers was exploited for developing a transformer-based VCO with low phase noise at 17 GHz [107]. A suggested design included a core VCO at 9 GHz followed by a frequency multiplier to achieve 17 GHz operation. Furthermore, a three-winding transformer design was suggested, serving as a resonator circuit and providing AC coupling between bases and collectors of VCO connected in cross-coupled fashion. In typical LC VCO designs, AC coupling is achieved through series capacitors which interact with varactors and lower the tuning range; and varactors of LC tanks are biased through high value resistors which increase noise. With the proposed transformer-based coupling, the need for AC coupling capacitors and high value bias resistors for varactors can be eliminated, and low noise VCOs with wide tuning range may be possible [107]. 

## 7. Conclusions

Ongoing developments in wireless communication technologies focus on building passive components on a single chip along with other RF circuitry to realize integrated systems. Therefore, the main aim of this paper has been to provide a thorough review on the performance, modeling methods, design methodologies, as well as, fabrication processes for monolithic on-chip transformers to serve as a guideline for developing new transformer architectures with improved performance.

On-chip transformer being an important passive component for many RF circuits, greatly affects the response of the overall RFIC—hence, high performance on-chip transformers are usually required. CMOS technology, however, being a solution for integrated systems, restricts the fabrication of monolithic transformers to planar topologies, which can only provide moderate performance, due to substrate losses. Alternatively, high performance monolithic transformers can be realized using MEMS fabrication and post-processing techniques which enable an on-chip realization of complex, out of plane structures. 

Therefore, a potential area of further research within the realm of on-chip transformers includes the development of novel transformer designs, fabrication technologies and CMOS-MEMS process integration. Accordingly, the developments and continuous improvements at the component level (e.g., transformers) will be instrumental towards the realization of circuits and systems with a superior performance necessary for emerging millimeter wave applications.

## Figures and Tables

**Table 1:** Comparison of different on-chip transformers using interleaved planar layout.

	Turn Ratio *N*	*Q*_MAX_	*f*_SR_ (GHz)	Secondary Inductance *L*_S_ (nH)	Primary Inductance *L*_P_ (nH)	Mutual Inductance *M* (nH)	*k*	*G*_MAX_	Outer Dimension *D*_OUT_ (µm)
[36]	4:5	9.3	11.4	3.4	-	-	0.7	0.77	280
[36]	4:5	8.3	15.5	2.9	-	-	0.7	0.74	196
[36]	4:5	3.1	4.5	3.4	-	-	0.7	0.53	280
[36]	4:5	2.22	5.4	2.9	-	-	0.69	0.46	196
[30]	3:2	-	16	1.42	2.53	1.25	0.66	0.79	202
[30]	3:2	-	13.6	1.28	1.45	1.28	0.66	0.85	254
[4]	5	-	-	9.75	0.39	1.482	0.76	-	400
[64]	3:3	-	-	-	-	-	0.83	0.575	367
[64]	3:3	-	-	-	-	-	0.90	0.576	367
[66]	4:4	9.86	9.2	3.96	-	3.02	-	-	142
[66]	4:4	8.53	10.4	3.93	-	3.04	-	-	118

**Table 2:** Comparison of different on-chip transformers using stacked layout.

	Turn Ratio *N*	*Q*_MAX_	*f*_SR_ (GHz)	Secondary Inductance *L*_S_ (nH)	Primary Inductane *L*_P_ (nH)	Mutual Inductance *M* (nH)	*k*	*G*_MAX_	Outer Dimension *D*_OUT_ (µm)
[44]	4	-	-	0.41	2.98	-	0.41	-	210
[24]	5.59	4.25	-	10.52	0.34	1.31	0.70	0.58	100
[24]	1.32	5.92	-	3.54	2.04	2.49	0.93	0.738	100
[25]	1	-	-	12.90	12.90	12.87	0.99	0.79	100
[25]	5.68	-	-	0.40	12.90	1.08	0.48	0.44	100
[22]	1.9	-	-	2.43	8.78	4.11	0.89	-	0.92
[22]	1.57	-	-	4.83	11.94	7.26	0.96	-	124
[71]	5.8	9.05	-	-	-	8.07	0.67	-	-
[38]	1.94	-	-	1.06	3.99	1.47	0.71	-	180
[70]	1	-	-	2.86	2.86	-	0.45	-	52
[21]	1	-	30.8	0.431	0.695	0.4948	0.92	-	140
[68]	1	10	6	-	1	-	0.96	-	200

**Table 3:** Performance comparison of different on-chip MEMS transformers.

	Self-Inductance of Coil *L* (nH)	Self-Resonance Frequency *f*_SR_ (GHz)	Quality Factor *Q*	*k*	*G*_MAX_	*f* (*Q*_MAX_) (GHz)	Process Technology
[77]	28.6, 33.4, 35	0.11, 0.17, 0.10	0.5, 0.57, 0.34	0.79, 0.4, 0.87	-	0.06, 0.09, 0.06	Surface micromachining
[34]	28.6, 29.3	0.08, 0.064	0.61, 0.35	0.9, 0.97	-	0.055	Bulk micromachining and Wafer bonding
[83]	7.14	-	5.92	0.75	0.68	0.5	Metal-MUMPs
[11]	88	0.051	5.9	0.98	0.80	0.01	Through-silicon-via technology
[84]	210	-	-	-	0.75	-	Surface micromachining
[26]	10.7	5.5	8.8	0.60	0.76	1	Metal-MUMPs
[27]	0.5	21	11	-	0.7	10	Front-side mask less etching
[85]	1.03	-	26.3	-	0.85	10	Substrate removal underneath the device using deep-trench technique
[14]	2.5	14	14	-	0.87	7.5	Concave-suspending bulk micromachining process
[20]	8	13.5	8.1	0.6	0.64	5	Substrate transfer technique and patterned ground shielding
[32]	4.3, 9.6	6.5, 3.6	9.1, 6.9	0.61, 0.65	-	0.8, 0.6	Bulk micromachining
